# Impact of Immune Cells in the Tumor Microenvironment of Prostate Cancer Metastasis

**DOI:** 10.3390/life13020333

**Published:** 2023-01-26

**Authors:** Justin K. Messex, Geou-Yarh Liou

**Affiliations:** 1 Center for Cancer Research and Therapeutic Development, Clark Atlanta University, Atlanta, GA 30314, USA; 2 Department of Biological Sciences, Clark Atlanta University, Atlanta, GA 30314, USA

**Keywords:** prostate cancer, metastasis, tumor microenvironment, immune cells, therapy

## Abstract

Prostate cancer is the most prevalent type of cancer in senior American men. Currently, the five-year survival rate after the initial diagnosis of prostate cancer is close to 100%. However, it is also the second leading cause of cancer death in senior men due to the dissemination of prostate cancer cells outside of the prostate causing growth in other organs, known as metastatic prostate cancer. The tumor microenvironment (TME) plays a critical role in the development, progression and metastasis of prostate cancer. One of the major components of the TME contains various types of immune cells, often recruited by cancer cells to the cancer formation areas. The interactions among prostate cancer cells and the infiltrating immune cells affect the outcome of prostate cancer. Here, we summarize the mechanisms various infiltrating immune cells use to regulate prostate cancer metastasis and possibly lead to the development of treatment strategies. Furthermore, the information here may also give rise to preventative strategies that focus on targeting the TME of prostate cancer patients.

## 1. Introduction

Prostate cancer is the most prevalent type of cancer in males, affecting approximately 1 in every 8 men in their lifetime according to the new cases in 2022 reported by the American Cancer Society [1]. Furthermore, it also accounts for the second leading cause of cancer death in men. Although several factors such as family history, diet, environmental agents etc. are linked to prostate cancer onset, the major risk factors are heavily associated with age and race [2,3]. For example, the median age at diagnosis with prostate cancer in American men is 66 years-old [1]. African Americans have the highest incident and death rate of prostate cancer, which is two to three times higher, among other racial groups. In addition, African American men are more likely to develop more aggressive prostate cancer [4].

The current five-year survival rate after the initial diagnosis with prostate cancer is approximately 97% given that almost all prostate cancer patients are diagnosed with this disease at its early stage while the prostate cancer cells are still contained within the prostate glands. Although the five-year survival rate is high, it dramatically decreases in subsequent years if the prostate cancer is unmanaged for multiple reasons. First, due to prostate cancer’s slow growth, it may take decades for cancer cells to become more aggressive and grow outside of the prostate. Given that prostate cancer patients are diagnosed in their late 60s, surveillance may be a better option according to the patients’ health conditions, however, the lack of urgency may cause the patient to become relaxed with their doctor visits. Second, removal of the prostate via radical prostatectomy, although directly eliminating the prostate cancer cells, may lead to urinary incontinence (being unable to control urine) and erectile dysfunction. Furthermore, prostatectomy may significantly reduce levels of testosterone [5] which is crucial for libido, bone density, muscle mass and strength, fat distribution and production of red blood cells and sperms in men [6,7]. Therefore, many men elect to not have the procedure. Third, there are no clinical tools which can predict when the prostate cancer cells may migrate outside of the prostate if under active surveillance and/or when the patients develop resistance to hormone deprivation therapy, a treatment intended to slow down the prostate cancer growth in the prostate. Once prostate cancer cells spread outside of the prostate, cancer metastasis, the five-year survival rate is dramatically reduced to 31% in 2020 [8] and it is referred to as incurable. The most common sites of the metastasized prostate cancer include the liver, bones and lymph nodes. It is believed that the local environment of these organs favors the migrated prostate cancer cells to re-root and grow there.

Although family history is one of the risk factors for prostate cancer [3,9,10], to-date, there are no known genes, such as oncogenes and tumor suppressor genes, responsible for causing prostate cancer. However, much of the research conducted on prostate cancer points to the tumor microenvironment being the key to prostate cancer development, progression and dissemination given that tumor microenvironment is dynamic and responds to the external factors such as diet, infections/pathogens and lifestyles. These factors provide a pro-inflammatory environment which is essential not only to prostate cancer onset [11], but also to all other types of cancer development [12,13,14].

## 2. Tumor Microenvironment

The tumor microenvironment (TME) is essential for tumor development, progression and eventual metastasis. Various secreted factors, surrounding blood vessels, rearrangement of the extracellular matrix and even immune cells all contribute to a constantly changing TME and tumor progression. The complex rearrangement of the ECM, the new formation of blood vessels and the constant communication between the various cells of the TME is orchestrated by tumor cells. Behind this crosstalk is a vast array of cytokines contributing to inflammation, chemokines which attract necessary components for a complex TME and remodeling enzymes such as metalloproteinases (MMPs) necessary for ECM alterations [11,12,13].

The immune system, which consists of various types of immune cells, is essential to our health. These cells are responsible for fighting off foreign pathogens and infections that individuals encounter on a daily basis. However, these immune cells also contribute to the TME since they are frequently recruited by tumor cells to the tumorous areas [14]. The most notable and well-studied immune cells found in the tumor favoring the TME include macrophages which secrete proinflammatory cytokines known to contribute to tumor progression, neutrophils that secrete enzymes known for ECM remodeling, regulatory T cells suppressing immune response as well as contributing to cytokine secretion, and mast cells secreting cytokines known to enhance migration. All together these cells work in unison to enhance tumor progression and metastasis.

## 3. Immune Cells to Modulate Prostate Cancer Metastasis

As mentioned previously, the immune cells of the TME play a major part in prostate cancer progression and metastasis. These well-studied infiltrating immune cells in the prostate include macrophages, neutrophils, T cells, B cells and mast cells. We summarize how each specific type of immune cell modulates prostate cancer metastasis below. A comprehensive understanding of their mechanisms, interaction with cancer cells and contribution to the prostate cancer TME will provide insight into future therapeutic treatments which may alter the cancer metastatic TME to a cancer-eliminating one which may benefit metastatic prostate cancer patients.

### 3.1. Macrophages

Macrophages are known for the critical role they play within the innate immune response. These phagocytic cells ingest foreign material in a nonspecific manner and present the foreign antigen on its surface in order to elicit a more direct and specific response from other cells found in the adaptive immune response. Although macrophages are critical for guarding our body from harmful pathogens and infections, they are also well-known for their contributions in cancer progression and metastasis [15,16,17,18]. As the most abundant cell within the tumor microenvironment, macrophages modulate cancer progression and metastasis in multiple ways including enhanced cellular migration, invasion and epithelial-mesenchymal transition (EMT).

Tumor associated macrophages (TAMs) secrete pro-inflammatory cytokines such as CCL5 and CCL2 which greatly affect the progression of prostate cancer and prostate cancer metastasis [19,20]. Both CCL5 and CCL2 have been shown to enhance the migratory abilities of prostate cancer cells ultimately contributing to their migration to other parts of the body including to the bone, the most common site of prostate cancer metastasis. In vitro studies using the exogenous addition of CCL5 to culture human prostate cancer cell lines DU145 and PC3 showed elevated migratory and invasive abilities of cancer cells. In addition, CCL5 treatment also produced larger size and quantity of cancer stem cells [19]. Furthermore, CCL5 expression was significantly increased in prostate cancer tissue samples and was correlated with higher Gleason scores [19]. Similarly, co-culture of prostate cancer cells with macrophages showed enhanced migratory abilities while cytokine analysis revealed CCL2 as the most abundant cytokine expressed by TAMs [20,21]. While STAT3 pathway activation led to upregulation of CCL2 and induction of EMT in prostate cancer cells [20], CCL5 was also found to activate STAT3 signaling [19]. Thus, it suggested that STAT3 signaling plays a role in the regulation of oncogenic pathways in cancerous cells and immune cell invasiveness. In vivo, PC-3 xenografted SCID mice injected with THP-1-derived TAMs showed a significant increase in bone metastasis while CCL5 knockdown blocked bone metastasis from prostate cancer, suggesting CCL5 as a major contributor to prostate cancer migration [19]. Androgen receptor (AR) knockout mice showed higher incidence of metastatic prostate tumors and higher corresponding levels of CCL2 [20] while other studies have shown that AR knockout causes a significant increase in MMP9 which has been indicated for its role in cancer metastasis [22]. CCL2 expression has also been shown to induce secretion of CCL22, a chemokine well-studied for its role in promoting cell migration of prostate cancer [23]. Another study reported that CCR2, the receptor for the CCL2 ligand, was increased significantly in prostate cancer cells when treated with CCL2 resulting in elevated cell migration of prostate cancer cells [21]. Furthermore, treating these cells with CCR2 antagonists severely impeded prostate cancer cell migration [21].

**Figure 1:**
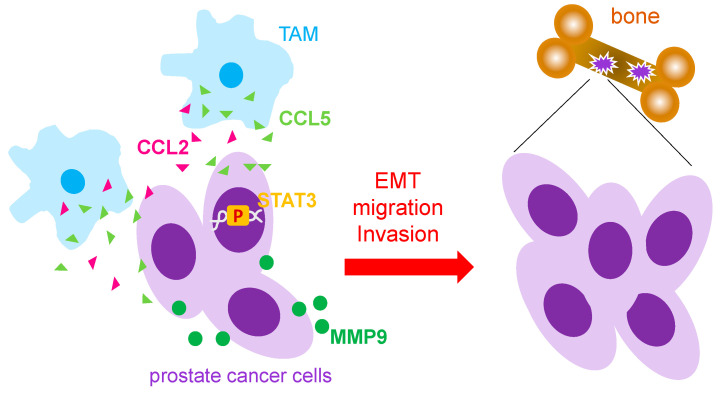
Mechanism that TAMs used to promote prostate cancer metastasis. Tumor associated macrophages (TAMs) have been reported to secret CCL5 and CCL2 to increase prostate cancer metastasis through activation of STAT3 and MMP9. EMT: epithelial-mesenchymal; MMP: metalloprotease; p: phosphorylation.

Depletion of TAMs in mice has also shown to be effective in reducing osteolysis in bone, the process of breaking down bone commonly seen in bone metastasis. TRAP5b, an enzyme known for its role in bone resorption, showed a significant decrease in macrophage depleted mice as compared to control. While macrophage depletion reduced the size of tumors found in the bone, induction of apoptosis of M2 macrophages also had the same effect [23]. Altogether, these findings demonstrate the importance of TAMs as it pertains to prostate cancer progression and metastasis (Figure 1). TAMs are capable of enhancing migration of prostate cancer cells via their secretion of various cytokines such as CCL5 and CCL2 while simultaneous activation of enzymes including TRAP5b grants access to new areas of the body, e.g., bones.

### 3.2. Neutrophils

Neutrophils, such as macrophages, work as first responders to foreign materials that enter our body. Once encountered, these white blood cells phagocytize the foreign materials while the components of the adaptive immune system organize their attack to generate a subsequent adaptive response. Much like the macrophages found within cancer areas, tumor associated neutrophils (TANs) have been reported in the cancer areas. However, in comparison to macrophages (TAMs), TANs are generally believed to have a lesser impact on TME due to their relatively short life. However, some research studies have started to focus on the potential role of TANs in tumor progression and metastasis and have discovered their influence is greater than originally thought [24,25,26,27,28].

Extracellular matrix (ECM) degradation is essential to cancer progression. This step is commonly achieved by increased expression and/or activity of metalloproteinases (MMPs) in the TME [29]. One of the well-studied MMPs is MMP9. Much of the research covering MMP9 secretion has heavily focused on its secretion from TAMs [30,31,32,33,34]. However, some current findings have begun to highlight the role TANs play in MMP9 secretion to promote cancer progression and metastasis. Neutrophils have been shown to secrete significantly higher levels of MMP9 compared to TAMs [35]. It has been demonstrated that the immediate amount of MMP9 secreted from neutrophils was equal to the total amount of MMP9 secreted in 30 days from macrophages [35]. On the other hand, some studies have shown the importance neutrophils play in trying to prevent prostate cancer progression [36]. For example, tissue samples from patients with bone metastatic prostate cancer showed an abundance of neutrophils proximal to the prostate cancer cells. Within this area, an array of neutrophil extracellular traps (NET) formation which are intended to limit the spread of infection by neutrophils was significantly increased, suggesting that TANs attempt to prevent prostate cancer metastasis [36]. Meanwhile, the C4-2B and LNCaP xenografted mice with neutrophil depletion had larger prostate cancer due to an increase in tumor volume as compared to control mice. Furthermore, depletion of TANs through its specific neutralizing antibody such as Ly6G reduced the cancer growth in the bones in a xenografted mouse model in which C4-2B prostate cancer cells were intratibial injected. However, in vitro studies showed that when cultured human prostate cancer cells C4-2B and LNCaP were co-cultured in the presence or absence of neutrophils, their cell growth was reduced via activation of caspases. The interpretation between the results in vitro and in vivo was that as the tumor progresses over time, neutrophil cytotoxicity begins to diminish which allows prostate cancer cells to effectively evade neutrophils cytotoxic effects [36]. These findings may provide an opportunity for future treatment strategies for patients suffering with bone metastasis resulting from prostate cancer through modifying neutrophils for their enhanced cytotoxicity and longer activation of tumor associated neutrophils.

Although results from the studies described above are contradictory, more research efforts are required to further reveal the role of TANS in prostate cancer metastasis and progression.

### 3.3. T Cells

T cells are a critical part of the adaptive immune response originating from the bone marrow. T cells are divided into multiple subtypes, including CD8^+^ T cells, CD4^+^ T cells and regulatory T cells. CD 8^+^ T cells, known as cytotoxic T cells, directly kill cells infected with a virus. On the other hand, CD4^+^ T cells, also known as T helper cells, contribute to the activation and expansion of memory B cells, another cell type of the adaptive immune response (see next section of B cells), as well as CD8^+^ T cells. Thirdly, regulatory T cells (Tregs) are vital for the recognition of foreign from self. When T cells are differentiated into their various subtypes, they undergo a process known as negative selection where cytotoxic T cells that attack host cells instead of pathogenic cells are eliminated. As a result, Tregs have the ability to inhibit or suppress the immune response to prevent healthy host cells from being destroyed.

Given that T cells are essential for a healthy immune system and that cytotoxic T cells, known as cancer eliminating cells are inactivated (loss of their tumor-killing abilities) during cancer development, certain subtypes of T cells have been reported to promote bone metastasis from prostate cancer. It has been shown that there is an increase in CD4^+^ T cells in the prostate tissue samples of prostate cancer patients in comparison to those from control [37]. Furthermore, chemokine CXCL9 secreted by the prostate cancer cells was shown to mediate CD4^+^ T cell recruitment to the prostate cancer regions, and CXCL9 blockage suppressed CD4^+^T cell migration towards the prostate cancer cells. It was found that CD4^+^ T cells were able to enhance prostate cancer cell migration and invasion abilities through downregulation of AR in the prostate cancer cells. The downregulation of AR expression in the prostate cancer cells resulted in recruiting more CD4^+^ T cells, providing a feedback loop for this interaction between the prostate cancer cells and CD4^+^ T cells enforcing invasiveness of the prostate cancer. Results from CWR22Rv1 xenografted orthotopic mice where cultured CD4^+^ T lymphocytic cell lines HH and Molt-3 were co-implanted into the anterior prostate and showed a significantly higher metastasis rate in the diaphragm as compared to the prostate cancer cell control group. Furthermore, immunohistology results of prostate cancer showed lower expression of AR and higher expression of MMP9 and FGF11. Growth factor FGF11 modulates cancer progression by regulating hypoxia signaling pathways. Moreover, elevated expression of FGF11 and increased infiltration of CD4^+^ T cells were detected in human prostate cancer tissue samples, verifying the link between infiltrating CD4^+^ T cells and prostate cancer metastasis via the identified mechanism as FGF11-AR-MMP9 axis in the prostate cancer cells [37].

Other studies have also revealed the importance of CD4^+^ T cells on chemotherapy resistance. It has been reported that following chemotherapy treatment with Docetaxel, a currently approved therapy for multiple cancers including metastatic prostate cancer, the numbers of CD4^+^ T cells in prostate cancer patients were higher in the tumor area [38]. Further analysis showed that co-culture of CD4^+^ T cells HH and Molt-3 with prostate cancer cells including C4-2 and CWR22Rv1 cells caused higher chemo-resistance to docetaxel-induced cell death in the prostate cancer cells. CCL5 was identified as a responsible cytokine secreted by CD4+ T cells for this high chemoresistance of prostate cancer cells. Treating prostate cancer cells with exogenous recombinant CCL5 resulted in a lower sensitivity of prostate cancer cells in response to docetaxel. Furthermore, blockade of CCL5 reversed this low sensitivity, indicating the effect of CCL5 in promoting prostate cancer cell survival in response to docetaxel. Finally, it was also shown that activation of STAT3 in the prostate cancer cells while co-culturing with CD4^+^ cell lines HH and Molt-3 mediated the chemoresistance of prostate cancer cells in response to docetaxel [38].

**Figure 2:**
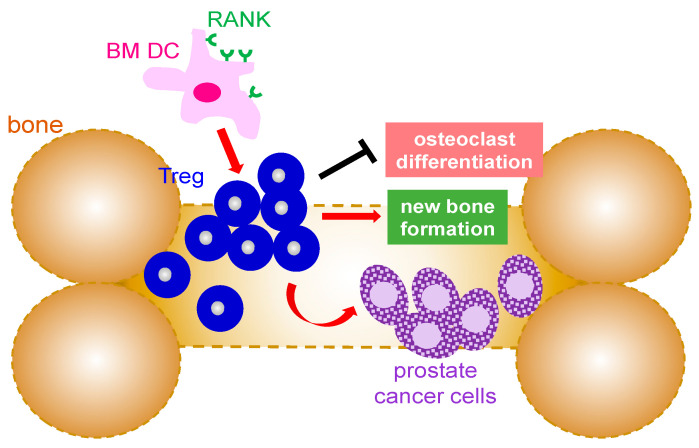
Mechanism that Treg cells are involved in bone metastases from prostate cancer. Infiltrating Treg cells promote the growth of the metastasized prostate cancer in the bone marrow environment as well as new bone formation by suppression of osteoclast differentiation. The expansion of Treg in the bone is mediated by RANK^+^ bone marrow dendritic cells. BM: bone marrow; DC: dendritic cell; Treg: regulatory T cells; RANK: receptor activator of NF-κB.

As mentioned earlier, Tregs are known for their immunosuppressive properties which make them ideal candidates for exploitation by cancer cells since cancer cells are self-cells and not foreign pathogens. Analysis of regulatory T cells in patients with prostate cancer showed a significant increase in Tregs in patients whose prostate cancer had metastasized to bone as compared to those with local prostate cancer [39,40]. These findings suggest that immunosuppression is increased at sites of metastasis (Figure 2). Studies to understand the cause of Treg cells migration into bone compartment in prostate cancer patients revealed that chemokine CXCL12, known for its role in stimulating tumor growth, was higher in prostate cancer patients with bone metastasis (Figure 2). Furthermore, inhibition of CXCR4, the receptor for CXCL12 ligand, significantly decreased prostate cancer cell migration [39]. In addition to CXCL12, other studies have also implicated cytokine CCL20 and its receptor CCR6 for their involvement in prostate cancer bone metastasis. It has been reported that CCR6 knockout mice had a better survival rate of bone cancer metastasized from prostate cancer [40]. Furthermore, blockade of CCL20 also resulted in higher survival of mice with prostate cancer bone metastasis. Of note, CCR6 knockout mice that had bone cancer metastasized from prostate cancer also possessed higher numbers of CD8+ cytotoxic T lymphocytes [40]. Another study demonstrated that Treg cells in prostate cancer patients with bone metastasis expressed a higher amount of cyclin genes with a simultaneous decrease in CDK inhibitors, suggesting Treg cells in bone marrow of prostate cancer patients undergo a pathological expansion to ultimately provide more immunosuppression [39]. Interestingly, dendritic cells (DCs) were shown to express RANK, the receptor for the RANKL cytokine which is known for its role in bone resorption [39,41,42,43]. Treg cell expansion was detected following the co-culture of RANK^+^ DCs with Treg cells whereas blockade of RANK reversed these effects. Furthermore, Treg cells were able to increase bone mineral density and bone mineral content in mice when inoculated with PC-3 prostate cancer cells in the tibia. Moreover, depletion of Treg resulted in a significant reduction in bone mineral density and bone mineral content. Altogether, it demonstrated a role of Treg cells in suppression of osteoclast differentiation [39].

### 3.4. B Cells

B cells are important for their role in the secretion of antibodies in the adaptive immune system. Each antibody secreted is specific for one antigen that identifies a specific pathogen the immune system is trying to eliminate. Once the antigen is encountered, the antibodies coat the outside effectively neutralizing the pathogen. Once the pathogen has been eliminated, B cells produce memory B cells to the specific pathogen which provide a quicker and more potent response to the same pathogen if subsequent infections occur. Although extensive research has shown B cells as a critical part in the adaptive immune response, so far, little is known about their role in cancer TME and how they contribute to cancer progression or elimination. Several studies in the pancreatic cancer field have demonstrated that the infiltrating B cells modulated pancreatic cancer development and survival in various ways including through their secreted IL-35, regulation of hypoxia as well as providing an inflammatory local environment etc. [44,45,46,47].

It has been showed that B cell density was significantly higher in prostate cancer regions than in adjacent normal prostate areas. However, it is unclear whether these infiltrating B cells are present in the prostate cancer regions for a specific purpose or not [48]. A study with the cohort analysis results from prostate cancer patients demonstrated that B cell infiltration in the prostate cancer region was more abundant in black males than white males and associated “improved recurrence-free survival” [49]. Unfortunately, this research was unable to scientifically explain this observation rather than the demonstration of the statistical analysis. Interestingly, another study implicated that higher amounts of infiltrating B cells in the prostate cancer tissues showed unfavorable prostate cancer patient outcomes [50]. Altogether, these contradictory study reports regarding the effect of infiltrating B cells on the prostate cancer TME as well as prostate cancer outcomes remain unsettled. Given that research studies addressing the role of B cells in prostate cancer and their mechanisms are limited, it presents an opportunity for a new and developing field.

### 3.5. Mast Cells

Mast cells are key players in the inflammatory response processes. They contain chemicals such as histamine and cytokines which are released during an immune response. In return, these chemicals cause an array of responses including vasodilation which allows other immune cells access to the infected area. However, the inflammatory process is also necessary for cancer progression.

**Figure 3:**
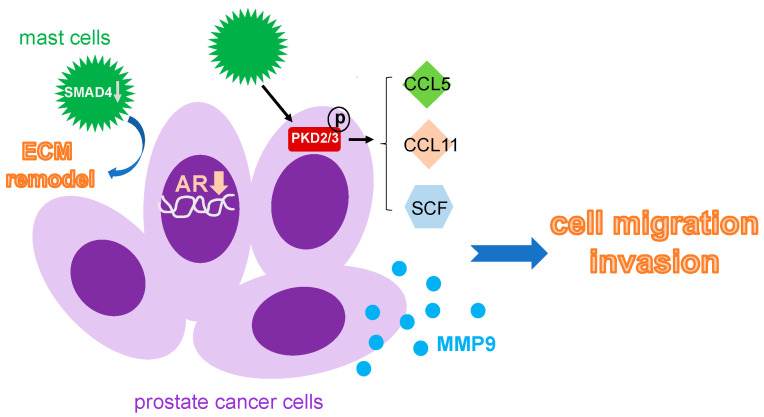
Mechanism that mast cells used to facilitate prostate cancer metastasis. Infiltrating mast cells can accelerate prostate cancer metastasis by decreasing AR expression pf the prostate cancer cell, increase CCL5, CCL11 and SCF through activation of PKD, and upregulate MMP9 expression. Moreover, the local ECM remodeling nearby is also controlled by mast cells via a downregulation of SMAD4. ECM: extracellular matrix; PKD: protein kinase D; SCF: stem cell factor.

Prostate tissue samples from patients with prostate cancer showed an influx of mast cells in the cancerous region as compared to normal regions [51]. Prostate cancer cells in the presence of mast cells had a significant increase in invasiveness as compared to those without mast cells. In addition, a reduced expression of androgen receptor was detected in the prostate cancer cells with the infiltrating mast cells. Furthermore, MMP9 expression was upregulated in cultured human prostate cancer cells including C4-2, C4-2B, LNCaP and CWR22Rv1 when co-cultured with mast cells [51]. These data suggested that infiltrating mast cells are able to increase the invasive ability of prostate cancer cells through a decrease in AR expression and an elevation of MMP9 in the prostate cancer (Figure 3). It has also been reported that mast cells have an effect on Protein Kinase D (PKD), a Serine/Threonine kinase playing a critical role in survival, growth and invasion of tumors. A significant increase in mast cells was correlated with an increased activation of PKD in prostate cancer cells from prostate cancer patients in comparison to those from normal individuals [52]. Furthermore, mast cells when co-cultured with human prostate cancer cell lines that express PKD2/3 promoted prostate cancer migratory abilities, implicating a role of PKD2/3 in associating with mast cell infiltration in the prostate cancer. Results from the migration assays showed that mast cell migration was mediated via various chemokines including SCF, CCL5 and CCL11 secreted by PKD2/3 expressed-prostate cancer cells. When PKD was knocked down through the siRNA technique, it suppressed mast cell migration. Moreover, this migration effect of mast cells can be rescued by addition of exogenous recombinant chemokines SCF, CCL5 and CCL11. In the allografted subcutaneous RM1 mouse model for prostate cancer, treating mice with specific PKD inhibitor CRT0066101 resulted in a decreased mast cell recruitment and showed no gain in tumor size or cancer progression such as angiogenesis [52].

Other studies have shown mast cells affect various genes that are associated with poor outcomes for patients with prostate cancer. Using the mast cells isolated from tumor and non-tumor regions of prostate tissue from patients with prostate cancer for a comprehensive gene expression array revealed that mast cells found within the tumor regions expressed genes related to pro-tumorigenic functions including tumor growth, differentiation, metastasis and immune escape. These genes included ARG2, ANXA2 and TIMP1 [53]. Furthermore, SMAD14, a key tumor suppressor gene, was downregulated in mast cells found within the tumor regions. When SMAD14 was overexpressed in cultured mast cell line HMC-1 for Proteomics analysis, a total of 148 proteins altered in the HMC-1-SMAD14^+^ cells were detected as compared to control mast cells and some of the target proteins have been associated with tumorigenesis, immune regulation and especially enrichment of proteins that are indicated in ECM disassembly. In addition, when culturing cancer associated fibroblasts (CAFs) with mast cell HMC-1 conditioned media, it rendered the ECM more conducive for tumor growth and metastasis [53].

Recruitment of mast cells have been reported to lead to prostate cancer cell resistance to docetaxel treatment through inhibition of docetaxel induced-prostate cancer cell apoptosis. It has been shown that the p38-p53-p21 signaling axis which is key to control the chemotherapy sensitivity of cancer cells was increased in prostate cancer cells in the presence of mast cells. As a result, it caused the prostate cancer cells to become resistant to docetaxel-induced cancer killing effect [54]. Furthermore, p38 inhibition through its pharmaceutical inhibitor partially restored prostate cancer cell sensitivity to docetaxel. So does inhibition of p53 as well as p21. In C4-2 subcutaneous xenografted mice, C4-2 cells pre-treated with mast cells showed more resistance to docetaxel treatment than those untreated, and they produced bigger tumors in volume and weight in vivo. Subsequent experiments also showed that prostate cancer cells including C4-2 and CWR22Rv1 in the presence of mast cells were more resistant to radiotherapy [54].

## 4. Conclusions

Although the five-year survival rate of prostate cancer after initial diagnosis is 97% when cancer cells are contained within the prostate, the chances of cancer progression and metastasis that lead to cancer death still remain relatively high. Many factors contributing to this progression involve the immune cells found within the TME as discussed previously. The secreted factors from these infiltrating immune cells in the prostate cancer TME such as cytokine CCL2 and CCL5 from TAMs, and metalloprotease MMP9 from TANs and mast cells, and inactivation of cytotoxic CD8^+^ T cells which can be mediated by Treg all work in unison to maintain the tumor’s ability to grow, migrate and invade. Since metastatic prostate cancer poorly responds to immune checkpoint inhibitors, known as immune cold cancer [55], it is of most importance to thoroughly understand the components of the TME and use the obtained knowledge to convert the pro-metastatic TME to an anti-metastatic one as a treatment strategy. The other alternative strategy would be to prevent the localized prostate cancer cells from metastasizing to other organs by keeping the TME under control as non-metastatic TME.
